# Shifting Practices Toward Recovery-Oriented Care Through an E-Recovery Portal in Community Mental Health Care: A Mixed-Methods Exploratory Study

**DOI:** 10.2196/jmir.7524

**Published:** 2017-05-02

**Authors:** Deede Gammon, Monica Strand, Lillian Sofie Eng, Elin Børøsund, Cecilie Varsi, Cornelia Ruland

**Affiliations:** ^1^ Center for Shared Decision-Making and Collaborative Care Research Oslo University Hospital Oslo Norway; ^2^ Norwegian Centre for E-health Research University Hospital of North Norway Tromsø Norway; ^3^ Department of Psychiatry Blakstad Division of Mental Health and Addiction Vestre Viken Hospital Trust Asker Norway; ^4^ University of Oslo Faculty of Medicine Oslo Norway

**Keywords:** recovery, eHealth, mental health, psychiatry, user involvement, empowerment, working relationships, participatory research, secure email, e-recovery

## Abstract

**Background:**

Mental health care is shifting from a primary focus on symptom reduction toward personal recovery-oriented care, especially for persons with long-term mental health care needs. Web-based portals may facilitate this shift, but little is known about how such tools are used or the role they may play in personal recovery.

**Objective:**

The aim was to illustrate uses and experiences with the secure e-recovery portal “ReConnect” as an adjunct to ongoing community mental health care and explore its potential role in shifting practices toward recovery.

**Methods:**

ReConnect was introduced into two Norwegian mental health care communities and used for 6 months. The aim was to support personal recovery and collaboration between service users and health care providers. Among inclusion criteria for participation were long-term care needs and at least one provider willing to interact with service users through ReConnect. The portal was designed to support ongoing collaboration as each service user-provider dyad/team found appropriate and consisted of (1) a toolbox of resources for articulating and working with recovery processes, such as status/goals/activities relative to life domains (eg, employment, social network, health), medications, network map, and exercises (eg, sleep hygiene, mindfulness); (2) messaging with providers who had partial access to toolbox content; and (3) a peer support forum. Quantitative data (ie, system log, questionnaires) were analyzed using descriptive statistics. Qualitative data (eg, focus groups, forum postings) are presented relative to four recovery-oriented practice domains: personally defined recovery, promoting citizenship, working relationships, and organizational commitment.

**Results:**

Fifty-six participants (29 service users and 27 providers) made up 29 service user-provider dyads. Service users reported having 11 different mental health diagnoses, with a median 2 (range 1-7) diagnoses each. The 27 providers represented nine different professional backgrounds. The forum was the most frequently used module with 1870 visits and 542 postings. Service users’ control over toolbox resources (eg, defining and working toward personal goals), coupled with peer support, activated service users in their personal recovery processes and in community engagement. Some providers (30%, 8/27) did not interact with service users through ReConnect. Dyads that used the portal resources did so in highly diverse ways, and participants reported needing more than 6 months to discover and adapt optimal uses relative to their individual and collaborative needs.

**Conclusions:**

Regardless of providers’ portal use, service users’ control over toolbox resources, coupled with peer support, offered an empowering common frame of reference that represented a shift toward recovery-oriented practices within communities. Although service users’ autonomous use of the portal can eventually influence providers in the direction of recovery practices, a fundamental shift is unlikely without broader organizational commitments aligned with recovery principles (eg, quantified goals for service user involvement in care plans).

## Introduction

Mental health care policies for those with long-term care needs are shifting from a primary focus on symptom reduction toward partnership models and personal recovery-oriented care [1]. At the same time, public health policies are promoting broad-scale implementation of eHealth technologies to strengthen people-centered care and public health capacity [2]. Several developments in eHealth are relevant to recovery-oriented care in mental health, such as enabling people access to their own electronic health records [3], shared decision making [4], self-management [5,6], peer support [7,8], online patient-reported outcomes [9], and service user involvement in research [10,11].

A common denominator of these developments is a shift in “locus of control” from health care providers toward service users by increasing the transparency of care decisions, as well as facilitating the voice and resources of service users in their care. In contrast to biomedical approaches that focus mainly on reducing symptoms, recovery-oriented approaches support people in articulating and regaining control over progress toward personal well-being goals [1,12,13]. Conceptualizations of the holistic and multifaceted nature of recovery are evolving in interaction with related fields such as self-determination and strength-based approaches [14,15], and is sometimes referred to as paradigmatic in that it disrupts established practice norms, priorities, and professional skill sets [16,17]. Accompanying emerging frameworks and guidelines for recovery-oriented practices are efforts to identify meaningful outcome measures across cultures and contexts [18-20]. Considerable work still lies ahead in identifying active ingredients of recovery, for whom, and under what conditions [15,21].

It is within this evolving landscape that this study describes the use of a recovery-oriented eHealth (“e-recovery”) portal “ReConnect” in two Norwegian community mental health sites during a 6-month period (2015-2016). ReConnect was designed using participatory methods. The rationales for portal design, including our path toward recovery as the guiding framework, are described elsewhere [22] (note that ReConnect was called “PsyConnect” in this previous publication). In this study, we sought insights into the question: what uses evolve when an e-recovery portal is made available in community mental health practices and what role does it play in terms of shifting practices toward recovery-oriented care?

## Methods

### Design

This study had a participatory design [23] and used mixed methods [24] to explore uses of an Internet-based intervention designed to support recovery-oriented practices in mental health care for people in need of long-term mental health care. The intervention was studied for a period of 6 months in two separate communities. Heeding calls for service user involvement in research [25,26], this study was conducted in collaboration with a service user consultant.

### Setting

Norway has universal health care that is funded by the public as part of the through general and earmarked grants [27]. The municipalities are responsible for providing primary health care and social services, whereas the Regional Health Authorities provide specialist services (eg, acute wards, district psychiatric centers). “Communities” in this paper refer to both levels of care provided to residents of two municipalities in Norway. These communities differed in characteristics, thus providing an intended variation in context: a small rural community with approximately 5500 inhabitants within an area of 1493 km^2^ versus a large community on the outskirts of the capital with approximately 52,000 inhabitants within an area of 100 km^2^. Management in the two communities expressed commitments to policies promoting eHealth, user involvement, and collaborative practices. The largest community explicitly expressed commitments to recovery principles in policy and strategy documents [28].

### Inclusion Criteria and Recruitment

The two communities became involved in the project through prior contacts with the principal investigator (DG). Multiple service entities at primary and specialist levels of care, as well as local service user organizations within the two communities, received written information and verbal presentations about the study. This information included the project’s overall aim of gaining insights into user needs and how e-recovery might facilitate or undermine service user involvement in treatment and collaboration with providers. Service users interested in participating in the study needed to fulfill the following criteria: they had to be older than 18 years; had to have received mental health services for at least 6 months prior to inclusion; and needed expectations of requiring services at least 6 months forward, electronic ID (see subsequently), and at least one provider willing to interact with them through ReConnect. As an exploratory study, efforts were made to recruit a wide range of participants in terms of gender, age, health issues, and types of ongoing support.

### Ethics

The study was approved by the Regional Committees for Medical and Health Research Ethics in Norway and the Privacy Protection Committees at the participating sites. Participants signed an online consent form before inclusion in the study.

### Organizational Anchoring

Local steering committees were established in both communities and consisted of primary and secondary health representatives (both clinicians and authorities), information technology (IT) management, and service user representatives. Their mandate was to ensure access to necessary resources (eg, clinician time, IT support, localities for training), and that the project harmonized with ongoing activities. Two hours of group training and/or individual training were held within both communities for service users and providers initially and when requested during the study period.

#### The E-Recovery Intervention

ReConnect was introduced to participants to support ongoing mental health care and treatment—whatever that treatment may be and as they saw fit. The stated objective of the portal was to support service user involvement in care, service user-provider collaboration, and personal recovery.

**Figure 1:**
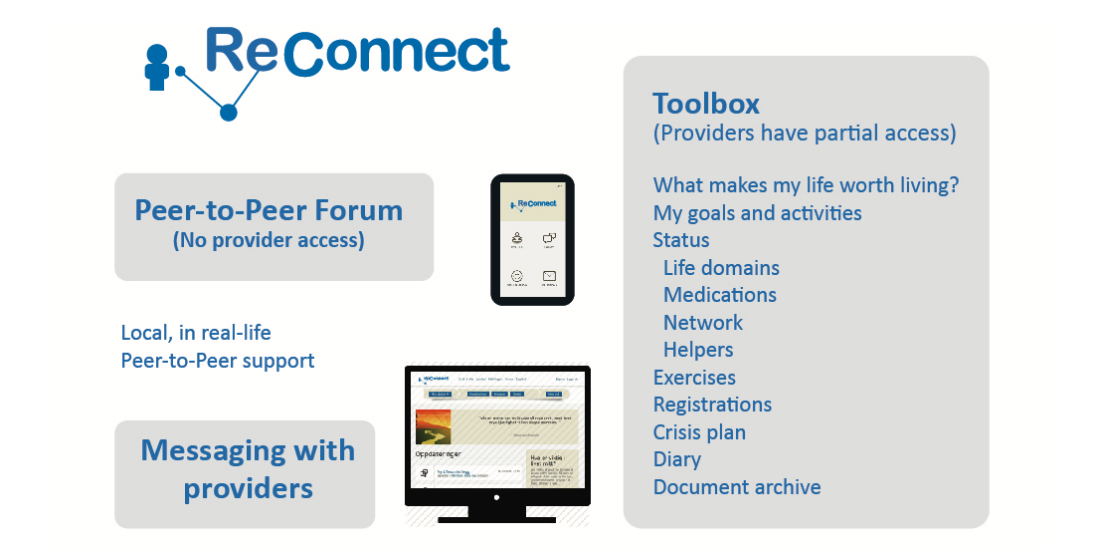
ReConnect portal.

As depicted in Figure 1, the portal consists of a toolbox, anonymous peer support discussion forum, and messaging with providers. Users log on using their electronic ID (eg, BankID), which is approved by the Norwegian government to allow patients to share personal health information in electronic and mobile apps. This ID is the same whether users log on to public services or online banking and is thus familiar to most Norwegians.

#### Toolbox

The toolbox can be likened to an interactive “workbook” and offers a wide range of resources that support service users in articulating and working with their personal recovery processes, including life domain status (eg, employment, social network, health), goals and activities, medications, network map, crisis plan, diary, and exercises (eg, sleep hygiene, mindfulness). Information related to patient rights and organizations were accessible either in the portal itself or through links. Simple help texts were available in all modules, as well as “good-to-know” texts (eg, how to formulate meaningful goals). Service users “owned” ReConnect in the sense that they determined how to use it to articulate aspects of “my life” and decide which providers had access to the user-generated content.

#### Forum

The anonymous peer-to-peer forum with service users from the two communities was moderated by LSE to ensure a safe and supportive environment.

#### Cafés

There were local real-life “ReConnect cafés” where service users could meet socially to discuss their uses of the portal and their own recovery processes.

#### Collaboration

Services users’ interactions with providers through ReConnect took place by messaging, by providers remotely accessing and commenting on the content of service users’ modules, or by sitting together and working with modules during consultations. The providers’ user interface included an overview of all their clients who used ReConnect, and they could remotely access the service users’ modules with some exceptions (eg, diary and forum). Thus, providers could follow the progression of service users’ activities (eg, homework in between consultations) and provide feedback as they saw fit.

Service users consented to using ReConnect exclusively for nonemergency purposes, and that ordinary channels had to be used for acute needs. Other than that, collaborative uses of ReConnect were determined by each respective service user-provider dyad as described previously. These were encouraged to clarify mutual expectations and routines, such as response time for messages (eg, daily, or once a week), absences (eg, holidays), and types of content (eg, providers might acknowledge receipt of messages with brief responses, but reserve therapeutic responses for consultations).

### Quantitative Data Collection and Analysis

After online registration and completion of the consent form, participants completed an online questionnaire containing questions about demographic characteristics, previous use of the Internet, and the following psychosocial measures:

1. Well-being was measured with the WHO-5 Well-being Index. The WHO-5 score can range between 0 to 100, in which 100 indicates the best possible well-being [29].
2. Anxiety and depression were measured with The Hopkins Symptom Checklist-25 (HSCL-25). The total HSCL-25 score can range between 1.0 and 4.0, and values greater than 1.75 indicate a need for help for the symptoms [30].
3. Patient activation was measured with the Patient Activation Measure (PAM). The score can range between 0 to100, and 100 indicates the best possible patient activation [31,32].
4. Satisfaction was measured with the Satisfaction Life Domains Questionnaire. The range of scores are 1 to 7, in which 7 indicates the best possible score [33].
5. Recovery was measured with the Recovery Assessment Scale (RAS). The range of scores are 1 to 5, and 5 indicates the highest recovery possible [34].

Data on portal use were extracted from the ReConnect user log on the server. All log-ins and uses per module were recorded in the system log.

Descriptive statistics were used to analyze questionnaire and system log data using SPSS version 21 (SPSS Inc, Chicago, IL, USA). Data are presented as medians and range for continuous variables due to skewed distributions and as proportion and percentages for categorical data. Demographics from the two communities are aggregated together to protect anonymity.

### Qualitative Data Collection and Analysis

Six focus group interviews were held, three with service users and three with providers, separately at each community after approximately 3 months of use. The focus group interviews were conducted by MS and LSE together. Questions were semistructured addressing current practices and expectations, experiences related to use, recovery processes, collaboration, and desired changes in the portal. A total of 12 cafés (six in each community) were moderated by LSE who facilitated discussions about use of ReConnect and about recovery processes. Both the focus group interviews and café sessions lasted approximately 1.5 hours and were audiorecorded. All focus group interviews were transcribed, whereas cafés were transcribed only when LSE’s notes indicated areas of interest to research analysis. Forum postings also served as data. Along with commenting on participant postings in the forum, LSE introduced topics for discussion relevant for recovery, such as hope, strengths, and citizenship. At times, authors DG, MS, and LSE discussed questions that LSE in turn posed to participants (eg, “Have any of you changed your way of collaborating with your provider through ReConnect and if so how?”).

Publicly available documents (eg, minutes of meetings) and personal communications regarding stakeholders’ actions following the study are referred to when reporting findings regarding the domain “organizational commitment” [35].

All transcripts from the focus groups, cafés, and forum were read through by authors MS, LSE, and DG, who added codes corresponding to the first two of Braun and Clarke’s six stages thematic analysis approach [36]. For this paper, DG searched for codes and quotes that illustrated Le Boutillier et al’s [35] four practice domains: (1) personally defined recovery, (2) promoting citizenship, (3) working relationships, and (4) organizational commitment. Because our aim was not to present themes based on an analysis of the total dataset (a focus of subsequent publications), but rather use the data to illustrate the practice domains, we present 3 to 5 illustrative quotes per practice domain. MS and LSE, who were most familiar with the informants and contexts from which quotes were extracted, reviewed the quotes in terms of credibility in illustrating the practice domains.

## Results

### Participants

#### Service User Characteristics

Of the 33 registered service users recruited, two withdrew right after recruitment. Twenty-nine answered the questionnaires and remained participants throughout the 6-month study period.

As presented in Table 1, participants had a median age of 44 (range 21-62) years, were predominantly female (86%, 25/29), single (69%, 20/29), and had an educational level of high school or less (69%, 20/29). In all, 31% (9/29) were employed either full-time or part-time, 28% (8/29) were on work assessment allowance, and 35% (10/29) were on disability benefits or retired. The service users reported a median of 2 (range 1-7) diagnoses (see Table 2 for elaboration on diagnosis).

**Table 1:** Demographic and illness characteristics among service users (N=29).

Characteristics		Participants
Age (years), median (range)		44 (21-62)
**Gender, n (%)**		
	Female	25 (86)
	Male	4 (14)
**Marital status, n (%)**		
	Married/cohabitating	6 (21)
	Divorced	3 (10)
	Single	20 (69)
**Education, n (%)**		
	Elementary/high school	20 (69)
	University/college	9 (31)
**Employment status, n (%)**		
	Full-time/part-time work	9 (31)
	Work assessment allowance	8 (28)
	Disability leave/retired	10 (35)
	Sick leave	1 (3)
	Student	1 (3)
**Site, n (%)**		
	Small community (5500 inhabitants)	14 (48)
	Large community (52,000 inhabitants)	15 (52)
Number of psychiatric diagnosis, median (range)^a^		2 (1-7)
**Psychosocial factors, median (range)**		
	Well-being	44 (0-80)
	Anxiety/depression	2.08 (1.24-3.68)
	Patient activation	56.40 (32.20-100)
	Satisfaction with life domains	4.11 (1.44-5.44)
	Recovery, total score	3.67 (2.33-4.50)

^a^ See Table 2 for list of diagnoses.

**Table 2:** Diagnoses reported by service users (N=29).

Reported diagnosis	n (%)
Depression	17 (59)
Panic anxiety	8 (28)
Generalized anxiety	8 (28)
Posttraumatic stress disorder	8 (28)
Phobic anxiety	5 (17)
Drug/alcohol addiction	4 (14)
Bipolar illness	4 (14)
Personality disorder	3 (10)
Schizophrenia	2 (7)
Obsessive-compulsive disorder	2 (7)
Schizoaffective illness	1 (3)
Mania	1 (3)
Other	4 (14)

The participants reported a median score of 44 (range 0-80) on the WHO-5 Well-being Index and a median score of 2.08 (range 1.24-3.68) on the HSCL-25 (anxiety and depression) indicating low well-being and an overall need for help with anxiety and depression symptoms (HSCL-25 cut off: 1.75). Their scores on patient activation, satisfaction, and recovery measures were in the middle of these scales, indicating room for improvements.

A total of 90% (26/29) used email daily or weekly, and 76% (22/29) used social media daily or weekly (see Multimedia Appendix 1 for more details of media use).

#### Health Care Provider Characteristics

Of the 27 participating health care providers, 14 worked in the municipalities and 13 worked in secondary level (DPC). They were predominantly women (89%, 24/27), 40 years or older (85%, 23/27), and most were nurses (11/27, 41%), social workers (5/27, 19%), and physicians (3/27, 11%). The remaining eight (28%) had different professions such as occupational therapist, psychologist, priest, interdisciplinary specialists, bachelor of psychology, or home care worker. There was a median of 19 (range 1-45) years since graduating from health professional education, and they had been working a median 10 (range 1-38) years within the field of mental health (Multimedia Appendix 2). All 27 providers used email and the majority used it daily (25/27, 93%) (see Multimedia Appendix 1 for more details on media use).

The dyads were highly diverse in terms of the diagnoses that service users reported and the professions reported by health care providers. Two service users had more than one participating provider.

### Types and Frequencies of Use

The median number of log-ins was 17 (range 1-151) (Table 3). Median number of messages sent was 2 (range 0-43). Modules not used by most participants could be frequently used and valued by one or two participants. This was particularly the case for crisis plan, network map, the medication list, and the diary. Some reported that having the options was valued, even though they had not used them yet.

**Table 3:** Usage of different components and activities in ReConnect during 6 months of access among service users (N=29).

Components and activities	Median (range)
Number of log-ins	17 (1-151)
“Good-to-know” article visits	3 (0-12)
Read article	0 (0-9)
Number messages views	14 (0-93)
Messages sent	2 (0-43)
Messages received	3 (0-40)
Crisis plan created	0 (0-1)
Diary post entries	0 (0-51)
Exercise visits	0 (0-9)
Forum Visits	21 (0-364)
Forum posts	3 (0-149)
Forum treads views	33 (0-508)
Medicine visits	1 (0-5)
Network map visits	2 (0-10)
Plan visits	10 (0-45)
Activity plan creation	0 (0-18)
Goal plan creation	0 (0-15)
Sub goal plan creation	0 (0-14)
Registration visits	6 (0-109)
Registration create	2 (0-128)
Update life domains assessment	1 (0-22)

#### Collaborative Use

Of the 27 health care providers, 19 (70%) answered secure messages from the service users. They answered a median of 6 (range 1-27) messages. (The system log failed to register types and frequencies of provider’s access to their service user’s module; therefore, we are unable to report this.) Both service users and providers reported that 6 months was too short of time to learn and optimally adapt their use of the various toolbox resources to their individual and collaborative needs. Examples mentioned included discovering relevant exercises after learning from peers in the forum, and that optimal use of the portal could differ when health was in a good versus a bad phase.

#### Forum

The forum was visited a median 21 (range 0-364) times per service user during their respective 6-month participation periods. During these 6 months, the service users posted 542 postings and viewed forum posts 1870 times in total (data not shown). The peer-moderator (LSE) initiated 167 of 542 postings (30.8%). Ten service users were active posters (>10 posts). No postings had to be removed due to inappropriate content. One service user reported obsessive use of the forum and together with his/her provider found ways to control use.

#### Cafés

In the 12 face-to-face gatherings, a total of 17 service users participated (range 3-9 per meeting). Several reported that becoming secure in the forum had been a prerequisite for mustering the courage to participate in the face-to-face cafés.

### Recovery-Oriented Practices

In the following, the experiences reported in focus groups, forum postings, and cafés by service users and providers are presented relative to Le Boutillier et al’s [35] four practice domains that were derived from a qualitative analysis of 30 international recovery-oriented practice guidance documents. These domains are summarized in Textbox 1. Although “practices” typically refers to actions taken by providers, we included the actions taken autonomously by service users through their use of ReConnect.

**Practice domains.**

**Supporting personally defined recovery**

- Practitioners focus on supporting personally defined recovery heart of practice and not as an additional task. Individuals are supported to define their own needs, goals, dreams, and plans for the future to shape the content of care. Individuality, informed choice, peer support, strengths focus, and holistic approach are contained in this practice domain.

**Promoting citizenship**

- The core aim of services is to support people who live with mental illness to reintegrate into society and to live as equal citizens. Citizenship is central to supporting recovery, in which the right to a meaningful life for people living with severe and enduring mental illness is advocated. Seeing beyond “service user,” service user rights, social inclusion, and meaningful occupation are grouped in this practice domain.

**Working relationships**

- Practitioner interactions demonstrate a genuine desire to support individuals and their families to fulfill their potential and to shape their own future. A therapeutic relationship, characterised as a partnership, is essential to supporting recovery in which hope is promoted.

**Organizational commitment**

- Organizations that support recovery orientation demonstrate a commitment to ensure that the work environment and service structure are conducive to promoting recovery-oriented practice. The organizational culture gives primacy to recovery and focuses on and adapts to the needs of people rather than those of services. Recovery vision, workplace support structures, quality improvement, care pathway, and workforce planning are included in this practice domain.

#### Supporting Personally Defined Recovery

Types of ReConnect uses that were particularly reflective of this practice domain were life domains, goal/activities, peer support, and the process of writing. Service users reported being helped in gaining an overview of their lives and becoming more conscientious of where they were headed and what kind of help they needed. For example, in a café discussion, one service user offered advice to another participant who was not getting the help he/she needed:

What I experienced as unbelievably positive for me was to sit down and divide up my life into the different life areas. It really increased my awareness. It became clearer for me where I stood, and where I wanted to head. I can really recommend it. You create for yourself a direction in life. At least that was my experience...[a lot of talking erupted in the group]...Maybe it would be easier for your helper to follow you up if she had something more concrete to work on...Maybe if you write it down it is easier for her to get a grip on what you need?

Some providers shared this assessment of the same modules. As one provider stated in a focus group:

The goal module has really helped. When he/she says “I wish I’d do more of this,” then I can put pressure on. When it’s written down in there as a concrete goal, then it kind of lights up a fire of sorts.

Another provider highlighted the value of service users’ creating descriptions of life domains and goals/activities in their own words:

It’s become a good way of structuring our work together. In a way, it’s clearer. What is his/her assignment or expectation of me?...It ensures that it is in fact his/her goal and not something I’ve written. One might think it’s the same, but the nuances in language can make a decisive difference in the actions we take.

The peer-to-peer forum and café gatherings were used to share experiences with the exercises (eg, mindfulness, strengths, self-created exercises) in support of defining one’s own direction. Among the many illustrative quotes in the forum:

I’ve just logged and made an exercise, I use all of them [exercises] except the ones for drug abuse. It’s a nice support for me when I’m working with myself. And it HAS helped me. From being isolated and very depressed to now getting out more. It’s helped to the point that I’m now working in a job 50%.

#### Promoting Citizenship

The peer support activities in the forum and cafés can be viewed as promoting community involvement (citizenship) in and of itself. Initially, peer support was established and maintained through the forum and subsequently expanded on and enriched through both the café gatherings and the focus group interviews. Friendships developed and plans were made for getting involved (eg, volunteering) in local activities. This included reflections on the role that community involvement can play in promoting health, and that providers need to support service users in this process. An example from a café discussion, that also illustrates the next domain (working relationships), is the following:

My mental health gets better when I help others. Be useful, do something meaningful, contribute to community. Those are things that helps your health and recovery. How can we get our helpers to support us in that kind of thing?

The issue that ignited the liveliest “community engagement” was at the end of the study when it was unclear whether ReConnect would be continued as a service within the two communities. This was evident in extensive forum discussions about how to influence community decision makers:

In any case, we’ve got to behave in the right way and talk to the people who are affected first, before we go to the newspaper, so we don’t step on the wrong people’s toes? But we can do this, right you guys? I hope you guys in [large community] are as enthusiastic as we [small community] are because we’re pretty fired up about keeping this service (six smilies)

A service user from the larger community responded:

If we’re going to the newspapers, we need to have a positive angle—not that we’re angry, or going to the barricades to fight, if we lose ReConnect. The smartest might be to go to the membership paper of the Norwegian Mental Health Association.

Eventually, two service users from the largest community contacted one of the project’s funding agencies who interviewed and photographed them for an article on their website.

#### Working Relationship

Dyad collaboration through ReConnect ranged from not at all to almost daily. This domain overlapped particularly with the first domain (supporting personally defined recovery) in that providers who supported service users in working with life domains, goals, and activities also reported having good working relationships. Collaborative uses included messaging, providers commenting the content of service users’ modules, and/or by sitting together and working with modules during consultations.

The life domains and goal/activity modules were frequently referred to by service users as helping collaboration with providers become more focused on their needs. The types of goals reported were typically short term (eg, per week) and very concrete. As one service user reported in a focus group:

Earlier it’s always been that [provider] asked me if I’d taken my medications, and then what openings there were in our calendars for my next consultation. Those two issues were what [provider] seemed mainly preoccupied with. Now with ReConnect we work more on my resources and goals—it can be as simple as managing to get through Christmas. How do I do it? Subgoals and activities can be buy the steak, avoid stress, get everything in the house, that type of thing—it was actually very useful to get ideas from another perspective—how to break down the problem...It really helps to break down the problem into smaller pieces.

Some service users expressed frustration that providers repeatedly told them how busy they were as an excuse for why they had not worked with them through ReConnect. For example, in a café discussion:

Why did [provider] agree to work with me through this tool if she never expected to do it? She should have just said no. You get so disappointed. That’s why it’s good to have each other [forum participants]—to call you my helpers. So we can share things.

This started a series of discussions about taking care not to overwhelm providers with messages or tasks, which caused one participant to react:

It’s completely understandable that constantly hearing how busy your helper is—I mean you don’t want to make life miserable for them. You don’t. But it’s just not right that us service users have to go around protecting our helpers.

These types of discussions in forum, focus groups, and cafés were typically accompanied by constructive suggestions for how to positively engage providers. One such exchange took place in a café discussion:

You’re right, it’s important for them [providers] to see that they’re useful to us—productive. The more specific we can be about what we need, the greater the chances that they’ll respond to us and our needs.

I think it was some smart advice from [another participant]. She gave her helper a clear assignment as to how to follow her up. I think several of us should do that. That’s how we create communication.

Some service users appreciated the flexibility that ReConnect introduced relative to in-person consultations that were sometimes described as unnecessary or unproductive. One service user, who received regular home visits, argued that flexibility could also benefit providers. As said in a focus group:

Maybe they don’t have to come so often if we can contact them [through the portal] when we’re working on something and need follow-up. Follow-up is what we need.

Providers, on the other hand, expressed concerned about pressuring service users to use ReConnect in ways that could be an added burden on them. For example, one reported in a focus group interview:

I’ve heard my service user say, “Unfortunately I’ve not answered, or done it” ...sort of like they have to apologize for not doing it [used ReConnect]. That’s why I’m kind of afraid of...it can be an extra burden on them...just following up things...Many are really vulnerable for stress.

This coincided with several providers who reported not wanting to put pressure on service users to use ReConnect, but that they were available if service users took the initiative.

#### Organizational Commitment

Most providers told of being committed to user involvement in care (a key recovery principle), whereas several reported barriers to committing to use of ReConnect as an ordinary service. Technical infrastructure-related barriers included inconveniences of having to log in with their private electronic ID (due to lack of integration with secure log-in system used by health care), multiple overlapping systems, and lack of integration with electronic health records.

Leadership in the large municipality initiated processes to address infrastructure barriers with the intention to implement ReConnect as a permanent service (minutes of meetings). Both the political and administrative leadership had committed to personal recovery—principles in all major policy and strategy documents [28]. This included a commitment to quantifying the extent of user involvement in individual care plans along with ambitious goals for an increase. ReConnect was viewed by leadership as enabling more effective progress toward policy goals (minutes of meetings). The smaller municipality also had user involvement as a goal, but without a specific approach or quantified goals. Here, the technical and financial commitments required to implement ReConnect were considered too great at the time.

Providers reported other barriers to committing to ReConnect. These included blurring lines between work and private life, lack of time allotted to answering messages, and concerns about the frequency and volume of written responses that might be expected by service users. Providers who appeared most positive toward ReConnect also reported being explicit about what service users could expect from them. One focus group participant, who described the portal as an asset to her work and benefit to service users, reported giving service users’ clear expectations:

I’ve told my clients that I answer messages Monday and Thursday mornings. That’s when they can expect answers from me. I need to have structure.

Another satisfied provider reported making agreements with service users that they would only respond to service users’ messages with brief responses to acknowledge receipt or clarify practical issues. More in-depth issues presented by service users would be acknowledged, then dealt with in their next consultation. Service users responded positively to these clarifications. Other providers valued saving time now that a service user had produced texts that could be taken directly into the statutory action plans. The fact that the service user also benefited from formulating and “owning” their own plans was referred to as “killing two birds with one stone.”

## Discussion

### Principal Findings

This descriptive and exploratory study sought to illuminate the question: how is an e-recovery tool used as an adjunct to ongoing community mental health practices and what role can it play in shifting practices toward recovery-oriented care?

The service users who used the portal became more involved in activities reflecting the first two of Le Boutillier et al’s [35] practice domains—personal recovery processes and citizenship—regardless of the practices of their provider. This was observable for the approximately 10 active forum posters and 17 café participants who also reported benefits similar to those reported in studies of online [37] and offline [38] peer support. Combining online and offline peer support with toolbox resources was an empowering common frame of reference for service users. Service users valued working more concretely on their personal life domains and goals, and in having a common vocabulary in discussing their experiences with peers. The opportunity to do so represented itself a shift toward recovery-oriented practices. Not only were service users offered a choice in terms of how they received mental health services, they could also choose to participate in defining their personal recovery processes and participate in community-promoting arenas. The service user who reported obsessive use of the forum, which was resolved together with his/her provider, was the only negative health-related experience reported among services users.

The positive role that the e-recovery portal played as a service separate from traditional services was highly dependent on the role played by the service user consultant (LSE) who moderated the forum and cafés. Although knowledge of optimal models for peer-run interventions is still evolving [12], communities who seek to promote recovery through similar portals will need to invest in similar types of expertise and role models for hope. Our experience suggests that the success of this role is closely linked with the acknowledgment of experiential knowledge as an asset within the community, in-depth familiarity of the principles of recovery, and the availability of discussion partners in health care when difficulties or dilemmas arise (LSE’s experiences will be elaborated on elsewhere). When sufficiently supported, such consultants with “lived experience” can contribute to mobilizing resources among service users and communities in ways that also can be valuable for improving the quality of health care services [12].

Use of the portal to augment treatment and its role relative to working relationships (the third practice domain) was less obvious. Dyad diversity, along with the nondirective way in which ReConnect was introduced to dyads (“use it as you see fit”), was reflected in highly diverse uses of the various portal resources. A total of 30% of providers never initiated or responded to messages, a source of frustration for service users. After 6 months, both service users and providers reported they were still discovering resources in the portal and adapting uses to their needs and preferences. This may partly be due to the shift in locus of control in that service users’ could now control the content of their own story and had a lowered threshold for linking documentation (eg, personal goals) to requests for follow-up. Both parties in working relationships can experience transitions of control as challenging [17,39], which likely adds to the time it takes to adjust.

Even if some dyads did not use the available resources in the portal to engage service users, the mere existence of the portal, and the dyads’ agreements to use it, inserted the topic of control into service users’ reports of their experiences in working with their provider. Some service users reported becoming empowered to make or request changes in the treatment they received, and that providers responded positively to these requests. However, such examples probably reflect good working relationships prior to use of ReConnect. Poor working relationships did not appear to improve through use of ReConnect, but rather were more clearly exposed as such. To explore how ReConnect can more systematically support working relationships in future studies, we have incorporated a short feedback-informed treatment measure to help dyads attend to the quality of their working relationship [40].

The largest community whose leadership had committed to recovery principles (ie, fourth practice domain) was also prepared to address the infrastructure barriers to implementing ReConnect as a permanent service. This may reflect greater financial and political resources compared to the smaller community, who did not make implementation of ReConnect a priority. More importantly, however, the largest community viewed ReConnect as a means for more effectively reaching quantified policy goals for user involvement in individual action plans [28]. This type of match between organizational values and the characteristics of the eHealth tool is an important success criterion for eHealth implementations [41]. Once an organizational commitment is in place, portals such as ReConnect can facilitate more rapid shifts in practices toward recovery, in addition to more rapid dissemination of new knowledge within communities.

### Limitations

We are not able to offer plausible explanations for the lack of men despite considerable efforts to recruit them, an issue which future service design studies need to address. Our opportunistic selection of quotes from superficially coded data to illustrate the four practice domains is not a balanced reflection of the experiences of participants. Thorough inductive analyses of participant experiences relative to collaboration and personal recovery are forthcoming. Nevertheless, we argue that the approach in this paper is justified in light of our aim of exploring the role such portals might play in shifting practices.

### Comparison to Prior Work

This study complements reviews of technically supported self-management interventions in general [6,42-44], as well as more specific recovery-oriented self-management interventions [45,46]. Our own scoping review of e-recovery found 20 studies of six recovery-oriented portals in five countries [47]. These studies have promising, but as yet no definitive findings related to enhanced shared decision making [48], strengths and resilient self-care strategies [49], social connectedness and empowerment [7,50], and patient-centered care [51,52], to mention a few. This study is one of the few to use a participatory approach with an exploratory design using mixed methods and, to our knowledge, the first to discuss e-recovery findings more systematically in light of a recovery framework. Several of the components in our portal are similar to other solutions with promising findings (ie, access to health records [44], shared decision making [42], and peer support [45,46]). Combining multicomponents into a single portal, as we have done, increases the challenge of sorting out active ingredients. At the same time, our study of how such a multicomponent intervention is used and influences ongoing practices helps pave the way for implementation of subsequent, more evidence-supported interventions in communities.

Based on this exploratory study, the following hypotheses can be proposed for future studies:

Personal recovery: people who have Internet-based tools that help them articulate what is important to them, coupled with providers who help operationalize “what is important” into concrete goals, are more likely to become actively engaged in their recovery processes than those without such tools and support.

Citzenship: e-recovery portals that combine Internet-based peer support with local in-real-life peer support are more likely to lead to community engagement than those who have access exclusively to one or the other.

Working relationships: working relationships via e-recovery are more likely to be effective if coupled with low-threshold feedback mechanisms that monitor the quality of such relationships than those without.

Organizational commitment: organizations with commitments to recovery principles are more likely to invest in and benefit from e-recovery portals than those without such commitments.

Although policy-pushes toward recovery and eHealth are so far largely based on values and resource constraints, e-recovery is unlikely to survive without evidence of its efficacy in helping people live fulfilling lives. Progress toward efficacy trials will need to build on more in-depth understandings of how digital resources interplay with recovery processes and for which service users, dyads/teams, and community contexts. Future research would benefit from recovery researchers joining forces with computer scientists in sorting out key recovery-oriented factors that can be co-created, boosted, tested in larger controlled trials, and implemented through digital innovations.

### Conclusions

The 24/7 availability of peer support and support for articulating personal goals in recovery processes represented itself a shift toward recovery-oriented practices within the participating communities. It is nevertheless the two practice domains, working relationships, and organizational commitment that are key to the more fundamental role that e-recovery portals can play in shifting practices toward recovery. Given organizational goals of monitoring service user involvement in care and the quality of working relationships, e-recovery portals can play a role in helping practices become more responsive to needs and aspirations as defined by service users.

## Appendix

Multimedia Appendix 1 Service users’ use of Internet and e-mail (n=29).

Multimedia Appendix 2 Health care provider characteristics and experience with use of Internet and e-mail of (n=27).

## Abbreviations

IT: information technology
PAM: Patient Activation Measure
RAS: Recovery Assessment Scale
