# Supplementary material for: Shifting Practices Toward Recovery-Oriented Care Through an E-Recovery Portal in Community Mental Health Care: A Mixed-Methods Exploratory Study
Source: J Med Internet Res. 2017 May 2;19(5):e145. doi: 10.2196/jmir.7524 (PMC5434256; doi:10.2196/jmir.7524)
Supplement: Multimedia Appendix 1 [file jmir_v19i5e145_app1.pdf]

Multimedia appendix 1: Service users use of Internet and e-mail (n=29)

**Use of Internet and e-mail**	n	(%)
**Sending/receiving e-mail**		
Daily	18	(62)
Week	8	(28)
At least every 14th day	2	(7)
At least once a month	0	(0)
Never	1	(3)
**Using internet banking**		
Daily	2	(7)
Week	14	(48)
At least every 14th day	10	(35)
At least once a month	2	(7)
Less often than a month	0	(0)
Less often	1	(3)
**Reading news on Internet**		
Daily	18	(62)
Week	7	(24)
At least every 14th day	1	(3)
Less often	1	(3)
Never	2	(7)
**Reading health info on Internet**		
Daily	3	(10)
Week	8	(28)
At least every 14th day	7	(24)
At least once a month	6	(21)
Less often	4	(14)
Never	1	(3)
**Participation in social medias/groups**		
Daily	19	(66)
Week	3	(10)
At least every 14th day	2	(7)
At least once a month	0	(0)
Less often	1	(3)
Never	4	(14)
**Communication through computer compared to face-to-face**		
Easier	11	(38)
More difficult	3	(10)
Nor easier or more difficult	11	(38)
Have no experience	4	(14)
