# Supplementary material for: Shifting Practices Toward Recovery-Oriented Care Through an E-Recovery Portal in Community Mental Health Care: A Mixed-Methods Exploratory Study
Source: J Med Internet Res. 2017 May 2;19(5):e145. doi: 10.2196/jmir.7524 (PMC5434256; doi:10.2196/jmir.7524)
Supplement: Multimedia Appendix 2 [file jmir_v19i5e145_app2.pdf]

Multimedia appendix 2. Health Care providers characteristics and experience with use of Internet and e-mail of (n=27).

**Characteristics an use of Internet and e-mail**		
**Age, n (%)**		
20-29 years	3	(11)
30-39 years	1	(4)
40-49 years	12	(44)
50-59 years	6	(22)
60 years or older	5	(19)
**Gender, n (%)**		
Women	24	(89)
Men	3	(11)
**Education, n (%)**		
Nurse	11	(41)
Physician	3	(11)
Psychcologist	1	(4)
Other[a]	12	(44)
**Year since finished health care education, median (range)**	19	(1-45)
**Years working within psychiatric care, median (range)**	10	(1-38)
**Years working at present employer, median (range)**	5	(0-33)
**Sending/receiving e-mail, n (%)**		
Daily	25	(93)
Weekly	2	(7)
At least once a month	0	(0)
Less than once a moth	0	(0)
Never	0	(0)
**Using net banking, n (%)**		
Daily	0	(0)
Weekly	18	(67)
At least once a month	9	(33)
Less than once a moth	0	(0)
Never	0	(0)
**Reading news on Internet, n (%)**		
Daily	22	(82)
Weekly	2	(7)
At least once a month	1	(4)
Less than once a moth	0	(0)
Never	2	(7)
**Reading health info on Internet, n (%)**		
Daily	7	(26)
Weekly	14	(52)
At least once a month	4	(15)
Less than once a moth	2	(7)
Never	0	(0)
**Participation in social medias/groups, n (%)**		
Daily	16	(59)
Weekly	5	(15)
At least once a month	1	(4)
Less than once a moth	1	(4)
Never	5	(19)
**Communication through computer compared to face-to-face, n (%)**		
Easier	4	(15)
More difficult	6	(22)
Nor easier or more difficult	16	(59)
Have no experience	1	(4)

[a] see table 2 for detailed list of professions
